# Transcriptome Sequencing and De Novo Assembly of Golden Cuttlefish *Sepia esculenta* Hoyle

**DOI:** 10.3390/ijms17101749

**Published:** 2016-10-22

**Authors:** Changlin Liu, Fazhen Zhao, Jingping Yan, Chunsheng Liu, Siwei Liu, Siqing Chen

**Affiliations:** Key Laboratory of Sustainable Development of Marine Fisheries, Ministry of Agriculture, Yellow Sea Fisheries Research Institute, Chinese Academy of Fishery Sciences, Qingdao 266071, China; liuchl@ysfri.ac.cn (C.L.); zhaofz@ysfri.ac.cn (F.Z.); yanjp@ysfri.ac.cn (J.Y.); lcs5113@163.com (C.L.); liusiwei_07@163.com (S.L.)

**Keywords:** cuttlefish, *Sepia esculenta* Hoyle, transcriptome sequencing, digital gene expression, early embryonic development

## Abstract

Golden cuttlefish *Sepia esculenta* Hoyle is an economically important cephalopod species. However, artificial hatching is currently challenged by low survival rate of larvae due to abnormal embryonic development. Dissecting the genetic foundation and regulatory mechanisms in embryonic development requires genomic background knowledge. Therefore, we carried out a transcriptome sequencing on Sepia embryos and larvae via mRNA-Seq. 32,597,241 raw reads were filtered and assembled into 98,615 unigenes (N50 length at 911 bp) which were annotated in NR database, GO and KEGG databases respectively. Digital gene expression analysis was carried out on cleavage stage embryos, healthy larvae and malformed larvae. Unigenes functioning in cell proliferation exhibited higher transcriptional levels at cleavage stage while those related to animal disease and organ development showed increased transcription in malformed larvae. Homologs of key genes in regulatory pathways related to early development of animals were identified in Sepia. Most of them exhibit higher transcriptional levels in cleavage stage than larvae, suggesting their potential roles in embryonic development of Sepia. The de novo assembly of Sepia transcriptome is fundamental genetic background for further exploration in Sepia research. Our demonstration on the transcriptional variations of genes in three developmental stages will provide new perspectives in understanding the molecular mechanisms in early embryonic development of cuttlefish.

## 1. Introduction

Cephalopods have great potential for aquaculture because of their fast growth rates, short life cycles, high food conversion efficiencies and high economic value [1,2,3,4,5]. The golden cuttlefish, *Sepia esculenta* Hoyle 1885 (Cephalopoda: Sepiidae) is one of the most important economic species distributed in the coasts of China, South of Hokkaido in Japan, Southwest coasts of Korea, and Philippines [6]. In China, the annual production of *S. esculenta* reaches the second place in that of squid in the world. However, due to a variety of reasons, such as overfishing and ocean environmental damage, the production of *S. esculenta* has sharply decreased since the 1980s [7]. In order to protect and exploit the germplasm resources of *S. esculenta*, China began to develop breeding technology since 2005 and has established certain scale aquaculture now.

However, the low survival rate of larvae in artificial hatching was limiting the industrialization of *S. esculenta* [8]. Though with a high hatching rate (more than 90%), the quality of hatched Sepia larvae was poor and the mortality rate was high, especially in the 5–8 days larvae at the opening stage (up to 80%). Effective techniques to improve the survival rate of early seedling cultivation were not available. Moreover, the low number of brood amount (about 2500 eggs) [9] and the short life cycle (one year) led to the amount of Sepia seedling unable to meet the demand of industrialization.

Fertilized eggs of Sepia went through several developmental stages, including cleavage stage, blastula stage, gastrula stage, organ forming stage, red-bead stage, heart beating stage before hatching out as healthy larvae [10]. Development of strategies to improve the survival rate of artificial hatching in Sepia cultivation required the understanding in biological processes of early embryonic development as well as the underlying molecular regulatory mechanisms. However, the genetic foundation and molecular mechanisms underlying the early embryonic development of Sepia remained elusive mainly due to the lack of genome information.

In this study, we presented a transcriptome sequencing data for gene model prediction. The assembled unigenes would provide the first resource for future molecular studies on biological and physiological mechanisms underlying the embryo development in Sepia. We also carried out a digital gene expression analysis among embryos at cleavage stage (CS), 5–8 days healthy larvae (HL) and 5–8 days malformed larvae (ML) for the identification of key genes and pathways that are involved in the regulation of embryo development in Sepia.

## 2. Results

### 2.1. Illumina Sequencing and Assembly

**Figure 1:**
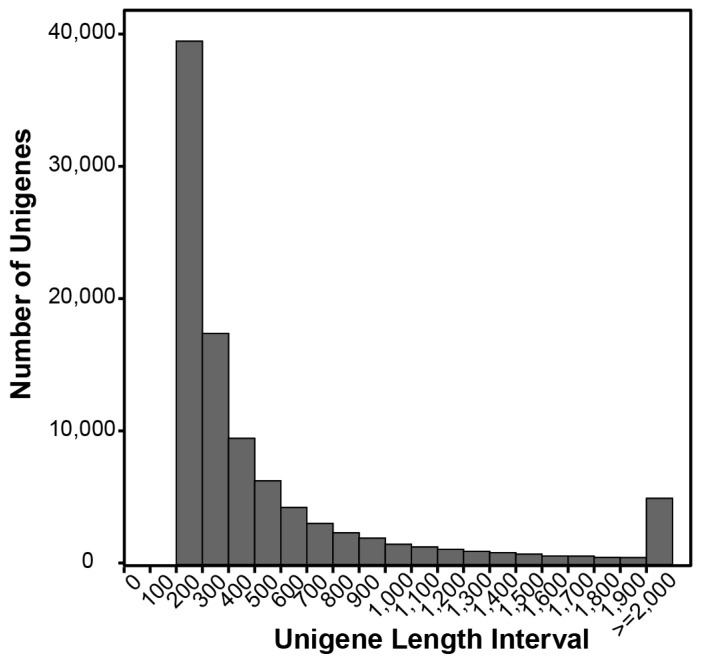
Length distribution of Unigenes.

To characterize the gene sets encoded by *S. esculenta* genome, especially genes involved in embryonic development, we collected embryos at cleavage stage, blastula stage, gastrula stage, organ forming stage, red-bead stage, heart beating stage, 5–8 days of normal larva and 5–8 days of abnormal larva as well for total RNA isolation. We generated 32,597,241 paired-end sequencing reads via mRNA-Seq on Illumina HiSeq 2000. Among them, 31,840,631 clean reads (97.7%) with a GC content at 40.11% were assembled de novo into 98,615 unigenes consisting of 61,246,386 bp. The length of these unigenes range from 200 to 19,292 bp, with the mean length at 621 bp and the N50 at 911 bp (Table 1). 32.4% unigenes were longer than 500 bp, and 14.1% were longer than 1000 bp (Figure 1).

### 2.2. Functional Categorization

To investigate the biological function of genes encoded by the *S. esculenta* genome, we first mapped the 98,615 unigenes yielded by the de novo assembly into the public database NCBI NR via blastx algorithm with an *E*-value threshold at 1 × 10^−5^. Only 19.18% of unigenes have at least one hit (Table 2). Among the genomes that harbored best Blast hits of *S. esculenta* unigenes in the NR database, 34.8% are Crassostrea, 5.6% are Branchiostoma, followed by the other 653 species.

**Figure 2:**
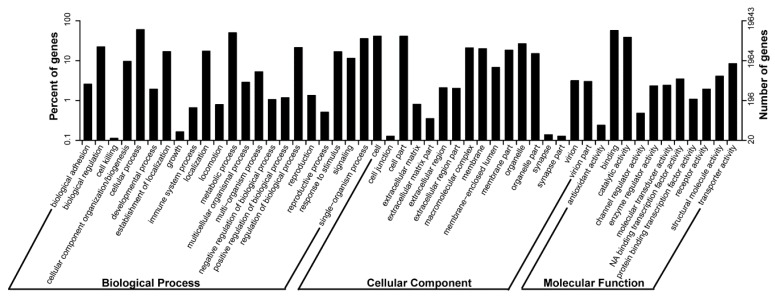
Functional classification of Unigenes by GO terms.

We mapped them to the Gene Ontology (GO) database via Blast2GO (v2.5) [11] to categorize the biological functions of Sepia unigenes. 19,643 unigenes matched to at least one GO term. Among them, 12,700, 12,339 and 8734 unigenes were assigned in biological process, molecular function, cellular component respectively (Table 2). In biological process, the most abundant functional groups were cellular process, metabolic process and single-organism process, etc. In molecular function, binding, catalytic activity and transporter etc. were the most highly represented functional groups, while in cellular component, more than 64% of GO terms are in groups of cell, cell part, organelle and macromolecular complex (Figure 2).

**Figure 3:**
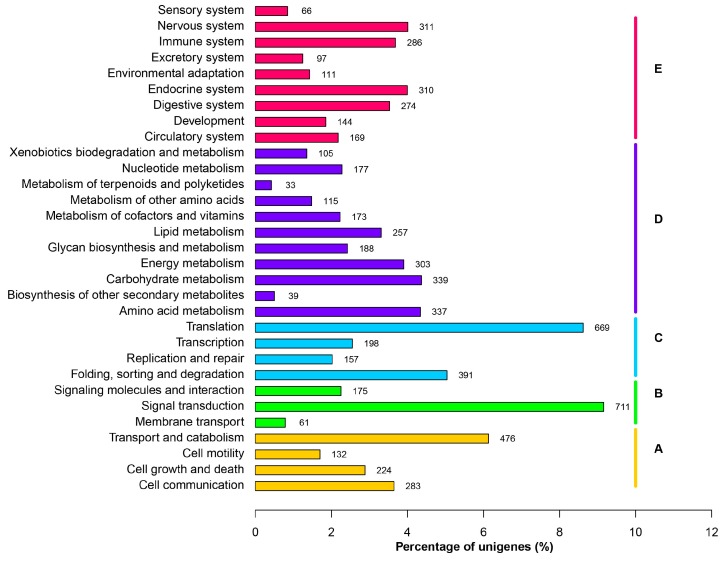
Functional classification of Unigenes by KEGG terms. The colors represent the five KEGG sections. The numbers at the end of each bar indicate the numbers of genes in each category. (**A**) Cellular process; (**B**) Environmental Information Processing; (**C**) Genetic Information Processing ; (**D**) Metabolism; (**E**) Organismal systems.

To better illustrate the physiological implications encoded by *S. esculenta* genome, we mapped unigenes into the referenced canonical pathways in KEGG database [12]. 4283 unigenes have at least one KEGG identifiers (Table 2). There were obviously more unigenes involved in signal transduction in environmental information process. For example, 272, 132, 126, 76, 64 unigenes were predicted to encode calmodulin in calcium signaling, classical protein kinase C in MAPK signaling, actin β/γ 1 in Hippo signaling, extracellular signal-regulated kinase 1/2 in MAPK signaling and integrin β 1 in PI3K-Akt signaling respectively. Among the metabolic pathways, genes related to carbohydrate, amino acid, energy and lipid metabolism were highly represented (Figure 3).

To comprehensively visualize the distribution of genes in individual metabolic reaction, we submit the information of KO IDs and number of genes related into iPath software [13]. There’re a significant expansion in dose of unigenes encoding the aldehyde dehydrogenase (NAD+) and fructose-bisphosphate aldolase (75 and 20 unigenes respectively) in carbohydrate metabolism, acetyl-CoA C-acetyltransferase (26 unigenes) in fatty acid metabolism, cytochrome P450 (49 unigenes) in steroid hormone biosynthesis and 4-aminobutyrate aminotransferase (30 unigenes) in amino acid metabolism (Figure S1).

### 2.3. Differentially Transcribed Genes among Cleavage Stage Embryos and Healthy Larvae

The key bottleneck of Sepia hatching is the extremely high rate of abnormality in embryo development leading to low survival rate of larvae. However, the genetic foundation and molecular mechanism of embryonic development in Sepia is currently unknown due to the limited genome information. To characterize genes involved in the early development of Sepia, we carried out a digital gene expression (DGE) analysis on embryos at cleavage stage (CS), 5–8 days old healthy larvae (HL) and 5–8 days old malformed larvae (ML). About 7.7, 9.4, 8.5 millions of sequencing reads with 100 bp length were obtained for CS, HL and ML samples respectively (Table 1).

**Figure 4:**
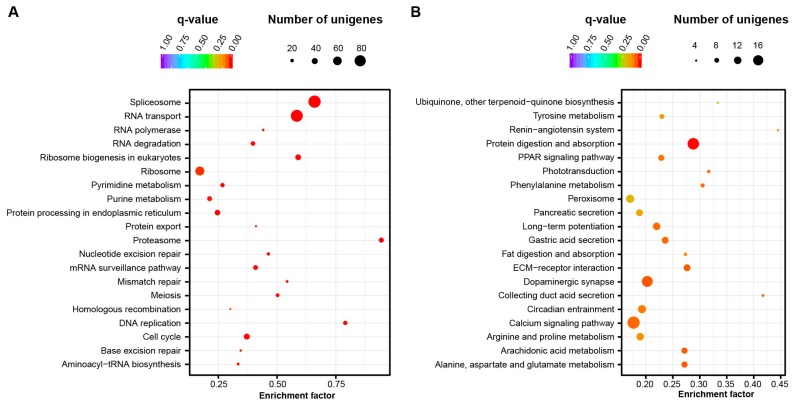
Enrichment of metabolic pathways exhibiting differential expression at cleavage stage and healthy larvae. (**A**) Enriched pathways with higher transcriptional level in embryos at cleavage stage; (**B**) Enriched pathways with higher transcriptional level in larvae.

Three thousand nine hundred twenty-four unigenes exhibited significant variation in transcription level between the two developmental stages CS and HL. Compared to healthy larvae, 1796 unigenes have higher expression level at cleavage stage. Among the functional groups they encoded, KEGG pathways related to cell proliferation, including DNA replication, spliceosome proteins, RNA transport, ribosome biogenesis, mismatch repair, meiosis etc. were enriched (Figure 4A). Among the 2128 unigenes showing increased expression levels in larvae compared to early developmental stage, genes involved in central nervous system morphogenesis and functioning (Arachidonic acid metabolism, circadian entrainment, dopaminergic synapse, long-term potentiation), protein related metabolism (amino acid metabolism including Tyrosine, phenylalanine, arginine and praline, alanine, aspartate and glutamate), phototransduction and tissue and organ morphogenesis related pathways (ECM-receptor interaction, peroxisome, PPAR signaling, calcium signaling), were enriched (Figure 4B).

**Figure 5:**
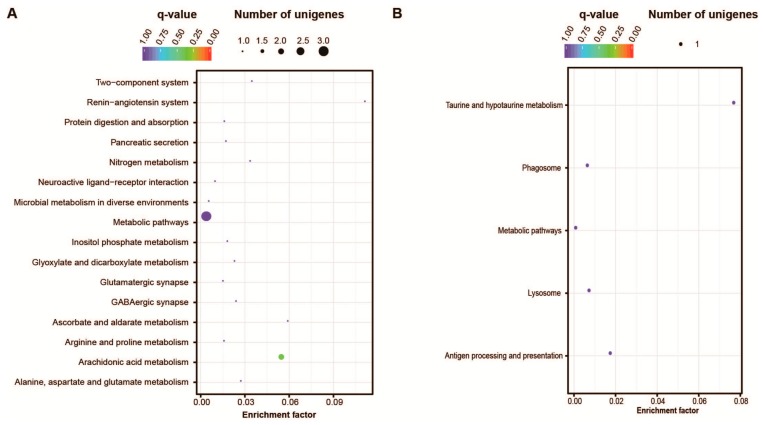
Enrichment of metabolic pathways exhibiting differential expression at healthy larvae and malformed larvae. (**A**) Enriched pathways up-regulated in malformed larvae; (**B**) Enriched pathways down-regulated in malformed larvae.

For the HL and ML embryos, only 46 genes exhibited significant variation in transcriptional level. Among them, 35 genes were up-regulated in malformed larvae. The KEGG pathways they were involved in were metabolic pathways, arachidonic acid metabolism, renin-angiotensin system and etc. (Figure 5A). Some of the genes were related to animal disease, such as the immunoglobulin binding factor *MSMB* (*β-microseminoprotein*), genes encoding the membrane attack complex/perforin protein, as well as cancer related gene *headcase* and *ARSB* (*arylsulfatase B*). Several genes functioning in organ development also exhibited significantly higher transcriptional levels, such as the gene encoding the Cysteine-rich secretory protein LCCL domain-containing 2 for promoting matrix assembly, gene encoding the angiotensin converting enzyme as one of the central component of the renin-angiotensin system and the neurite-promoting factor *S-crystallin*. We also found two proteinase genes, *trypsin* gene and a metalloproteinase gene *myosinase* I, were also up-regulated. However, among the 11 genes with decreased transcriptional level in malformed larvae, only one gene was functionally annotated. It encoded the lysosomal endopeptidase cathepsin L which was involved in the initiation of protein degradation (Figure 5B).

**Figure 6:**
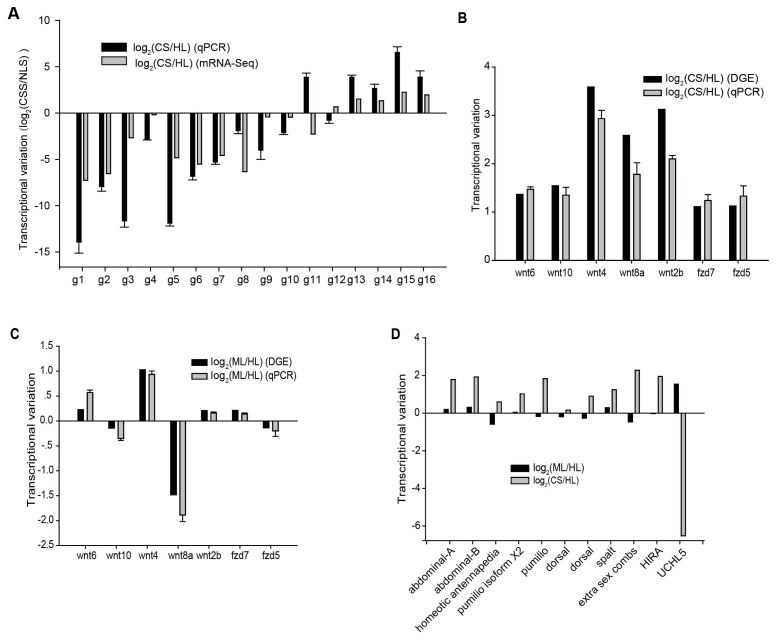
Expression variation of embryonic development related signaling pathways based on mRNA-seq data. (**A**) The transcriptional variation of 16 genes calculated from qPCR and mRNA-seq data; (**B**) The transcriptional variation of Wnt signaling genes between CS and HL; (**C**) The transcriptional variation of Wnt signaling genes between ML and HL; (**D**) The transcriptional variation of genes involved in other signaling pathway.

Besides, to investigate the reproducibility of our data, we performed quantitative RT-PCR on a set of selected 16 genes. Their transcriptional variations estimated by qRT-PCR were consistent with the ones referred from the digital sequencing data (Figure 6A).

### 2.4. Signaling Pathways Related to Embryo Development

To primarily explore the regulatory mechanism involved in the embryo development of Sepia, we identified the putative homologs of genes related to key signaling pathways using tblastn in the assembled gene sets of Sepia. In Sepia genome there were 13 genes encoding putative homologs of Wnt genes, including *Wnt1*, *Wnt2b*, two *Wnt4*s, *Wnt5*, *Wnt6*, *Wnt7*, *Wnt8a*, *Wnt8b*, *Wnt9*, *Wnt10*, and *Wls* [14,15]. Compared to healthy larvae, among them, *Wnt2b*, *Wnt4*, *Wnt6*, *Wnt8a* and *Wnt10* exhibited significantly higher transcriptional levels in cleavage stage. Especially *Wnt8a* and *Wnt2b* had a 6.0 and 8.7 fold of level respectively in cleavage stage. Moreover, in malformed larvae, the transcription of *Wnt8a* decreased. The key player in dorsal-ventral axis formation in amphibians, *Wnt11*, showed stable transcription, though *Wnt5* showed a weak decrease of expression in CS (Figure 6B). Wnt proteins bind to their cognate cell surface receptors to initiate the signaling process. Among the other 29 members involved in Wnt signaling identified in current gene models of Sepia, only *fzd5* and *fzd7* were up-regulated in CS and none of them showed significant transcriptional variation between ML and HL (Figure 6C).

Genes in several other signaling pathways regulating embryo development of animals were also identified, including three genes encoding Hox family transcriptional regulators (homeotic antennapedia protein, abdominal-A and abdominal-B), two genes encoding the Puf Family RNA-binding translational regulators (pumilio and pumilio homolog 2), two NF-κB family genes *dorsal-1* and *dorsal-2*, a Zn-finger transcription factor gene *spalt*, the Polycomb group (PcG) gene extra sex combs (*esc*) and the histone cell cycle regulator gene *HIRA*. Except homeotic antennapedia protein and the two dorsal genes, all the other 7 genes showed higher transcriptional level in CS than HL though no significant variations detected in ML. Ubiquitin carboxyl-terminal hydrolase L5 (UCHL5) was reported to be interacting with Smads and regulating TGF-β signaling. Interestingly, the gene *UCHL5* in Sepia was significantly up-regulated in ML than HL and exhibited almost undetected exhibition (Figure 6D).

## 3. Discussion

Sepia has captured people’s attention due to its critical role in the global fisheries. Many studies have focused on investigating the physiological characters, environmental adaptations and cultivation improvements in Sepia [8,16,17,18]. However, though several sequencing data records deposited in NCBI database, there’re no systematic dissection on the genome and transcriptome information of Sepia published [19]. The lack of genome information of Sepia has greatly blocked the exploration of genetic background of key physiological features of Sepia and seeking rational solutions on currently obstacles in artificial hatching and scaleful cultivation. In 2012, the Cephalopod Sequencing Consortium (CephSeq Consortium) proposed a sequencing strategy for several Celphalopod species, including *Sepia officinalis* which was a popular model organisms in neurobiological research and eco-evo-devostudies [20]. The fascination of scientists on Cephalopod, emphasizing their economic value, also indicated the urgent requirement of genome information in current Cephalopod research area.

Due to the large genome size and repeat-rich structure in Sepia genome, de novo sequencing and assembly would be technically challenging [21]. An optional sequencing strategy is the transcriptome sequencing to construct the functional gene models in genome. In this study, we have produced transcriptome sequencing data using illumina Hi-Seq 2000. The constructed gene models would provide the first knowledge for further biological studies. The number of assembled unigenes, 98,615, is a higher than the one in the other Cephalopod *Octopus vulgaris* (59,859 unigenes) [21]. The low number of unigenes in *Octopus vulgaris* might be due to the limited sampling tissues in central nervous system. The low match rate of *S. esculenta* unigenes in NR database might be due to the limited genome and transcriptome information of species that are closely phylogenetically related to *S. esculenta*. The other possible reason might be that the lengths of unigenes are too short to allow statistically meaningful matches.

The constructed gene models facilitate us to identify genes and signaling pathways that are reported to be involved in embryo development in invertebrate. The digital gene expression analysis among embryos at cleavage stage, healthy larvae and malformed larvae provided the first blueprint of regulatory mechanisms underlying Sepia development, and would also identify candidate targets that might play critical roles in Sepia development. This work will facilitate further functional characterization and rational genetic manipulation to improve the robustness of artificially hatching embryos. Many signaling pathways are known to play important roles in the embryo development of animals, such as the Hox family regulators, Wnt signaling pathway [14,15]. Wnt signaling pathway is one of the most important signaling pathways in regulating embryonic development in animals. It plays essential roles in regulating developmental processes including body axis formation, organogenesis through coordinating various cell behaviors such as cell proliferation, cell movement and stem cell maintenance. In this study, we investigated the transcriptional changes of key genes in Wnt signaling pathway in cleavage stage embryos and unhealthy larvae respectively compared to healthy larvae. The observation of *Wnt8a* with much higher expression in cleavage stage confirmed its maternal origin, and also indicated its important role in specifying the dorsal-ventral axis as in Zebrafish. The knowledge would be immensely helpful for exploring molecular targets and novel strategies to improve the survival rate of Sepia larvae in artificial hatching. The identification of most of key genes involved in multiple signaling pathways indicated that our sequencing sample mixture collected from embryos at a series of developmental stages was able to cover genes involved in embryo development and to provide a molecular resource for further functional studies.

## 4. Experimental Section

### 4.1. Sample Collection

Sepia esculenta Hoyle parents were captured by the cage net fishing during May to July 2013 from Qingdao Sea area (120°20′–120°38′ E, 35°74′–35°92′ N). They were kept in the lab for future research. The fertilized Sepia eggs were collected and incubated in ocean water at 18–25 °C. Larva began to take off the membrane and hatched out after 26–28 days. The developmental stages were determined under anatomical microscope on 3–5 randomly picked embryos after stripping their egg membranes [10]. About 200 embryos at cleavage stage, blastula stage and gastrula stage were collected respectively, and stored in RNAlater (Ambion, Austin, TX, USA) at 4 °C. For organ forming stage, red-bead stage, heart beating stage, 5–8 days of normal larvae and 5–8 days of malformed larvae, about 100 embryos were collected and stored respectively in the same way.

### 4.2. RNA Isolation and Illumina Sequencing

Total RNA was isolated using Trizol (Invitrogen, Carlsbad, CA, USA) from the above embryos and larvae individually following the Invitrogen’s instruction. The quality of total RNA was detected using both argarose gel and Agilent 2100 Bioanalyzer (Palo Alto, CA, USA). Only RNA samples with clear 18S and 28S rRNA bands on gel, as well as RIN > 7.0 were used for sequencing libraries preparation following instructions by Illumina (San Diego, CA, USA). Totally we had four libraries sequenced by Illumina HiSeq 2000. One was the paired-end 2 × 100 bp mRNA-Seq library. The RNA used was a mixed sample containing equal quantity of RNAs from all the eight developmental stages. The other three sequencing libraries were digital gene expression sequencing (DGE sequencing) for RNA samples from cleavage stage embryos, healthy 5–8 days larvae and 5–8 days of malformed larvae respectively. All the raw reads were deposited in NCBI SRA with accession number as SRP041769.

### 4.3. Transcriptome Assembly

Raw reads generated by HiSeq 2000 were filtered to remove reads containing adapter, reads containing poly-N and low quality reads via a in-house perl script (available upon request). Clean reads resulted were used to do de novo assembling using Trinity program with min_kmer_cov set to 2 by default and all other parameters set default [22].

### 4.4. Gene Annotation

cDNA sequences of Unigenes were aligned to the NCBI NR database via blastx (v2.2.28, *E*-value threshold at 1 × 10^−5^). Information of the top 10 hits of each unigene in NR was extracted via an in-house script (available upon request). Blasting against NT database (NCBI non-redundant nucleotide sequences) was performed using blastn. Annotation in Pfam (Protein family) database and Swiss-Prot (A manually annotated and reviewed protein sequence database) were performed using hmmscan. Annotation in KOG/COG (Clusters of Orthologous Groups of proteins) was performed via blastx. Functional GO annotation was performed using blast2GO program (v3.0) [11]. Metabolic pathway analysis was carried out though mapping to KEGG database via KAAS [12]. Unique KO IDs encoded by Sepia unigenes were extracted and submitted to iPath v2 [13], with the edge width standing for the frequency of each KO IDs (edge width = log_2_(Frequency + 1) × 5).

### 4.5. Differential Gene Expression Analysis

Raw data of DGE sequencing were filtered using a perl script. Expression levels of unigenes were estimated by RSEM (rsem-1.2.0) for each sample [23]. The read counts generated were then adjusted by edgeR program package through one scaling normalized factor [24]. Differential expression analysis of two samples was performed using the DEGseq (2010) R package (v1.12.0) [25]. Unigenes with adjusted *q*-value less than 0.005 and the fold change more than 2 were considered as significantly differentially expressed unigenes. Statistical enrichment of differential expression genes in KEGG pathways were performed by KOBAS software (v2.0) [26].

### 4.6. Quantitative Real Time PCR

To verify the DGE data, we collected Sepia embryos under the same growth conditions with the ones used for DGE sequencing. Embryos at the each developmental stage were divided into three groups as biological replicates. Reverse transcription was carried out using M-MLV reverse transcriptase (Promega, Madison, WI, USA) with oligo (dT) as primers. PCR was perfomed using SYBR Green I on ABI prism 7500 PCR. The primers of the 16 genes and Wnt signaling pathway genes, as well as the reference gene actin, were as follows: Wnt6-F: CTGGCACTCTGACAACGCTA, Wnt6-R: ATTGGATTGTGGTGGGCAGT; Wnt4-F: CCGTTCCATTTTGCCGCTAC, Wnt4-R: CTGAACAGAGGAACGCGAGA; Wnt2b-F: AGAAGGCACCGGGTTAATCG, Wnt2b-R: CTCGACCGCAGCACATGATA; Wnt8a-F: GCGTTACACGAAGGCAATCC, Wnt8a-R: CGTTTGCATTCCCGGTTCAG; fzd7-F: TCATGCAATCCCTCCGTGTC, fzd7-R: GATGTTCTCTCCGACGCACA; fzd5-F: TGAACACTACTGAACGGCCC, fzd5-R: AACGGCAGGAACAATCGTCT; actin-F: GCGCGTCTTTCCTGTCAAAG, actin-R: GCTTGTATTTCTCCTGACGCC. g1(comp56552_c0)-F: GGCATTTCCGGTCAAACCAC; g1-R: ACGTGATGGTGACACTGGAC; g2(comp50180_c1)-F: GCAAAGGATGTTGGACTGCAC; g2-R: ACCGCCAAAAGCAGAGGTAA; g3(comp50185_c0)-F: TGCAAGCCAGAGGTATCCAC; g3-R: TCGTTGACACCCCTCATCATC; g4(comp26063_c0)-F: CGAGAAGAGAAACTCCCGCA; g4-R: TTGCACTTGGACGACCTGAA; g5(comp56616_c0)-F: AGTCTTCGGCTTGAGTCTGC; g5-R: AAAAGCTCGTCTCACCCAGG; g6(comp56572_c0)-F: ATCCCGTGCTTAACTGCCAT; g6-R: GTTCGCACTTTGTCGCTGTT; g7(comp56581_c1)-F: TGCGATGACTGAACGACAAG; g7-R: GACTGTCCAGCTGACTTGGAAT; g8(comp50077_c0)-F: TCCTTGAATCGGGTTGGCTC; g8-R: CACTGCTTTGGGCTTCAACC; g9(comp26064_c0)-F: TCCACCATATTCTCGGCCCAT; g9-R: ATCGAGTGGCTTGGGTATTGA; g10(comp56590_c0)-F: ACCGTGCCATCAATAGGTGT; g10-R: AAAAGAGTGATTGGCTCCACA; g11(comp49425_c0)-F: TCCCCGATTGTGATGACGAC; g11-R: AAAGTCAGCAGTTCCGAGCA; g12(comp26068_c0)-F: TGCATGAATCGGCCACAAGA; g12-R: GCAGCCACTTACGCATCATC; g13(comp56749_c0)-F: AGTGGCACAACCAAGTCCAA; g13-R: TGATCCTGCCGATTGCTTGT; g14(comp56691_c0)-F: AATCCGAACGAGGAACCAGC; g14-R: CATCTCGTTCCCCTGCATGA; g15(comp56654_c0)-F: CACAACCAGCAGACCAGACA; g15-R: CCCAAGGAGTATGCCACAGG; g16(comp26151_c1)-F: TGGTCGAACTCTCGTGGTTG; g16-R: AGTTTACGGTGTGTGGCGAT.

## Figures and Tables

**Table 1:** Summary of sequencing raw data and assembly.

Statistics of Sequencing Data	Numbers
Raw reads of mRNA-Seq	32,597,241
Clean reads of mRNA-Seq	31,840,631 (97.7%)
Unigenes assembled	98,615
Total length of unigenes (bp)	61,246,386
Ave. length of unigenes (bp)	621
N50 length of unigenes (bp)	911
Max. length of unigenes (bp)	19,292
Min. length of unigenes (bp)	201
Raw reads of the three DGE samples	7,671,799 ^a^/9,439,043 ^b^/8,512,259 ^c^
Clean reads of the three DGE samples	7,562,335 ^a^/9,305,149 ^b^/8,376,805 ^c^

^a^: cleavage stage (CS); ^b^: healthy larvae (HL); ^c^: malformed larvae (HL).

**Table 2:** Functional annotation of Sepia unigenes.

Databases	Number of Unigenes	Percentage (%)
Annotated in NR ^1^	18,921	19.2
Annotated in NT ^2^	2720	2.8
Annotated in KEGG ^3^	7761	7.9
Annotated in SwissProt	15,078	15.3
Annotated in PFAM ^4^	19,150	19.4
Annotated in GO	19,643	19.9
Annotated in KOG ^5^	10,547	10.7
Annotated in all databases	1516	1.5
Annotated in least one database	25,462	25.8

^1^: NR, NCBI non-redundant protein sequences; ^2^: NT, NCBI nucleotide sequences; ^3^: KEGG, Kyoto encyclopedia of genes and genomes; ^4^: PFAM, Protein family; ^5^: KOG, Clusters of orthologous groups of proteins.

## Supplementary Materials

Supplementary materials can be found at www.mdpi.com/1422-0067/17/10/1749/s1.
