# Supplementary material for: Transcriptome Sequencing and De Novo Assembly of Golden Cuttlefish Sepia esculenta Hoyle
Source: Int J Mol Sci. 2016 Oct 22;17(10):1749. doi: 10.3390/ijms17101749 (PMC5085775; doi:10.3390/ijms17101749)
Supplement: Supplementary file 1 [file ijms-17-01749-s001.pdf]

Supplementary Materials: Transcriptome Sequencing and De Novo Assembly of Golden Cuttlefish *Sepia esculenta* Hoyle

Changlin Liu, Fazhen Zhao, Jing-ping Yan, Chun-sheng Liu, Siwei Liu and Siqing Chen

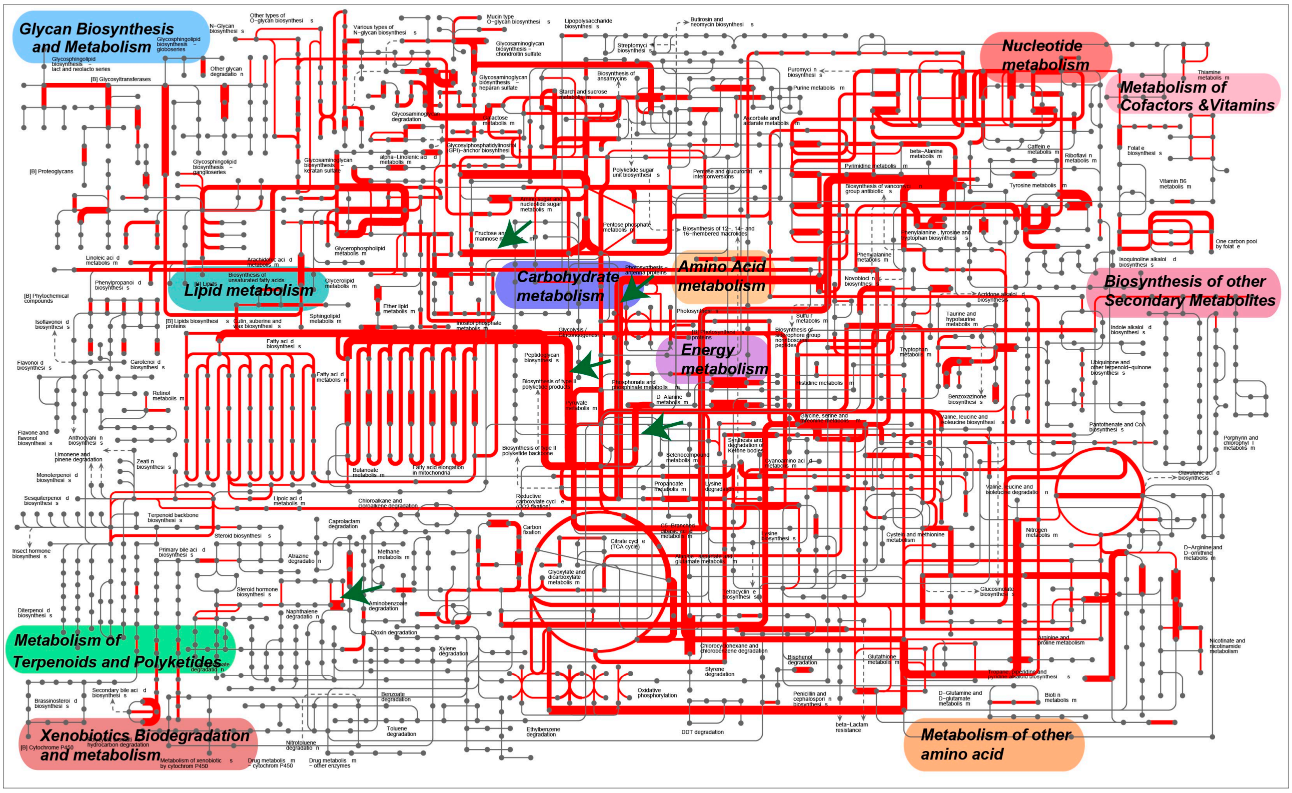

Figure S1. Metabolic pathways in KEGG encoded by unigenes and gene dose of individual KO ID. The metabolic enzymes encoded by expanded copies of unigenes were indicated with green arrows.
